# Effects of neuromuscular training on athletes physical fitness in sports: A systematic review

**DOI:** 10.3389/fphys.2022.939042

**Published:** 2022-09-23

**Authors:** Saddam Akbar, Kim Geok Soh, Nasnoor Jazaily Mohd Nasiruddin, Marrium Bashir, Shudian Cao, Kim Lam Soh

**Affiliations:** ^1^ Department of Sports Studies, Faculty of Educational Studies, Universiti Putra Malaysia, Seri Kembangan, Malaysia; ^2^ Department of Sports Studies, Faculty of Education Studies, Universiti Putra Malaysia, Seri Kembangan, Malaysia; ^3^ Department of Nursing, Faculty of Medicine and Health Science, Universiti Putra Malaysia, Seri Kembangan, Malaysia

**Keywords:** neuromuscular training, physical fitness, skill-related fitness components, health-related fitness components, athletes

## Abstract

**Objective:** This review study aimed to assess the impact of neuromuscular training (NT) on athletes’ physical fitness in sports.

**Methods:** Three independent reviewers conducted a literature search in various databases: EBSCOHOST, PubMed, WOS, Mendeley, Scopus, ProQuest, Science Direct, additional references, and Google Scholar. The methodological quality was examined using Lubans’ predetermined methods, and data that included trials were excluded.

**Results:** This review included 18 well-conducted systematic studies from 144 relevant publications. These studies were reviewed and have been given a score of 6. Medium-risk studies were scored 3 or 4, while low-risk studies were scored 5 or 6. None of the studies had a high-risk bias. The NT intervention revealed that balance (*n* = 10) was the main characteristic of physical fitness that was evaluated, followed by agility (*n* = 6), muscular strength (*n* = 4), speed (*n* = 5), endurance and muscular power (*n* = 2). Subsequently, most studies used an intervention such as plyometric and strength training exercises to improve agility, balance, and muscular strength among athletes.

**Conclusion:** This review implicated that (NT) focuses on exercises that enhance motor skills which aid athletes in moving their bodies according to their situational needs. The athletes’ slower and faster directions influence their agility, muscular strength, and balance, essential for player performance. It is recommended for future research to investigate the effects of neuromuscular training (length of 12-weeks, frequency of 3 days per week and 90-min duration) on physical fitness components (coordination, reaction-time, flexibility, cardiovascular fitness, cardiorespiratory fitness and body composition) that are essential for all ages of male and female athletes in all sports.

## 1 Introduction

An athlete’s success relies heavily on the combination of ability, tactical, technical, physical fitness, and psychological individualities (Smith, 2003; Granacher et al., 2016). Physical fitness is vital in determining an athlete’s competitive abilities (Kariyawasam et al., 2019). Physical fitness is influenced by various factors, including genetics and the environment. A lot of genetics and environmental factors have an impact on human physical performance. Extremely hot or cold climates strain the exercising individual, striving to stabilize their internal body temperature (Cech, 2011). Furthermore, some characteristics that affect fitness, including body size and muscle fiber composition, are influenced by inheritance. Inherited variables may influence physical activities (Bouchard and Perusse, 1994). According to assessments from heritability investigations on sport-related factors, speed-power, endurance and strength capacities all have a genetic cause (Coelho et al., 2016; Pickering et al., 2019; Guilherme et al., 2021), which could be explained in part by the genetics of muscle fiber specialization (Hamada et al., 2000; Lewis et al., 2015). Professional youth soccer players’ physical features vary depending on their maturity level (Murtagh et al., 2018), and strength being even more important in mature professional youth players than in immature elite youth soccer players (Murtagh et al., 2018). Furthermore, it is uncertain if genetic changes may reflect differences in strength and speed among professional players and various phases of development actively involved (Murtagh et al., 2018). Case-control studies in primarily older players have made up most of the past genetics research in soccer (Egorova et al., 2014; Gineviciene et al., 2014). However, in contrast to cross-sectional genotype and phenotype association research, such investigations are of partial significance to the utilized practitioner, even if they do not reveal the link concerning bodily functional abilities and individual heritable variants. Further, there are few genotype and phenotype studies on athletes (Micheli et al., 2011; Pimenta et al., 2013; Coelho et al., 2016), it is uncertain whether the influence genetic diversity has on elite soccer players’ strength, speed, power and quickness. To the researcher, no study has been done on the interaction between neuromuscular training and heredity.

Neuromuscular training (NT) is a strength and fitness training method that combines sport-specific and fundamental movements, including resistance, balance, core strength, dynamic stability, agility exercises, and plyometrics, to improve skills and health-related fitness (Myer et al., 2011). Furthermore, NT aims to enhance speed, reaction speed, agility, coordination, and endurance among athletes (Matin et al., 2014). NT, which has grown in popularity in recent years, are used to prevent injuries and improve the performance of athletes by regaining leg power, strength, and balance after an injury (Hewett et al., 2005; Batrakoulis et al., 2018; Canli, 2019).

The NT improves nerve-muscle control while increasing the stability of functioning joints (Yoo et al., 2010; Gonçalves et al., 2020; Mahdieh et al., 2020). Furthermore, NT has been proven to influence the sensitivity and reactivity of the central nervous system and improves the power of athletes by targeting motor units and coordinating motor units and increasing muscle activation. These enhancements resulted in skillful movements and significantly improved agility, balance, muscular strength, muscular power, and cardiorespiratory endurance among individuals (Fort-Vanmeerhaeghe et al., 2016). The NT also focuses on promoting functional joint stability by improving athletes’ neuromuscular control and has been proven to significantly enhance their VO_2_ max (Schneider et al., 2006). Numerous studies have focused on plyometrics, strength, and NT on general and soccer-specific performance in youth (Michailidis et al., 2013; Fort-Vanmeerhaeghe et al., 2016; Dhawale, 2020). Neuromuscular training aims to improve neuromuscular control and increase functional joint stability. It is anticipated that addition balance training in these programs will enhance the coactivation of the muscles around joints, increasing joint stiffness and active joint stability. Furthermore, it could change biomechanical injury risk factors, such as raised knee valgus during landing exercises in female high school basketball players (McLeod et al., 2009). The NMTP aims to improve athletes’ ability to manage their center of mass during dynamic activities (Myer et al., 2006). NMTP significantly improved measures of athletic performance in female athletes, particularly female basketball players, as well as biomechanical factors.

Most studies have examined the effects of INT on adolescent motor performance, and the results are extremely positive. For instance (Faigenbaum et al., 2011; Sugimoto et al., 2014; Faigenbaum et al., 2015) found significant improvements in several motor performance indicators following 8–10 weeks of INT (Sugimoto et al., 2014). demonstrated that high adherence to INT significantly increases the isokinetic strength of the hip abductors in 15-year-old female volleyball players, while (Faigenbaum et al., 2014) found significant gains in neuromuscular fitness and aerobic endurance measures following 8 weeks of INT in 7-year-old children.

It has been suggested that throwing athletes could benefit from increased neuromuscular control through neuromuscular training (Freeston et al., 2015). Neuromuscular training has been considered an effective treatment method to enhance the neurophysiological entity of the joints for coordinated functioning (Zech et al., 2009; Bscher et al., 2010). Neuromuscular training, defined as training that enhances unconscious motor responses by stimulating sensory signals and central mechanisms, leads to dynamic joint stability (Zech et al., 2009). This training improved dynamic joint stability and fine motor control by strengthening the synchronisation and synergy of the muscle activity pattern in cricket players (Lephart and Henry, 1996; Myers and Lephart, 2000; Guido Jr and Stemm, 2007).

Integrative neuromuscular training (INT) is a program that aims to improve health and skill-related aspects of physical fitness (Faigenbaum et al., 2011; Myer et al., 2011) that has been trending in recent years (Faigenbaum et al., 2011; Myer et al., 2012; Faigenbaum et al., 2014). The INT prioritizes muscular power, motor skill performance, and muscular strength (Myer et al., 2011; Fort-Vanmeerhaeghe et al., 2016). In addition, the program includes resistance training, plyometric exercises, and dynamic stability (Myer et al., 2011; Myer et al., 2012; Faigenbaum et al., 2015), which improves the athletes’ vertical jump. Most importantly, INT may act as injury prevention due to the strengthening, stretching, plyometrics, and balance exercises included in these training programs (Hewett et al., 2005).

Previous studies have demonstrated that NT alters neuromuscular risk variables, resulting in a significant increase in athletic performance and physical fitness. However, there is no systematic study of the NT program on skills and health-related fitness components (Michailidis et al., 2013; Gonçalves et al., 2020; Sinđić et al., 2021). Therefore, this aims to perform a comprehensive evaluation and synthesis of the scientific literature on the impact of NT on athletes’ physical fitness. Furthermore, this review is the first study to use a structured approach to the literature and to evaluate NT programs and fitness components in player performance.

## 2 Methods

### 2.1 Protocol and registration

**FIGURE 1:**
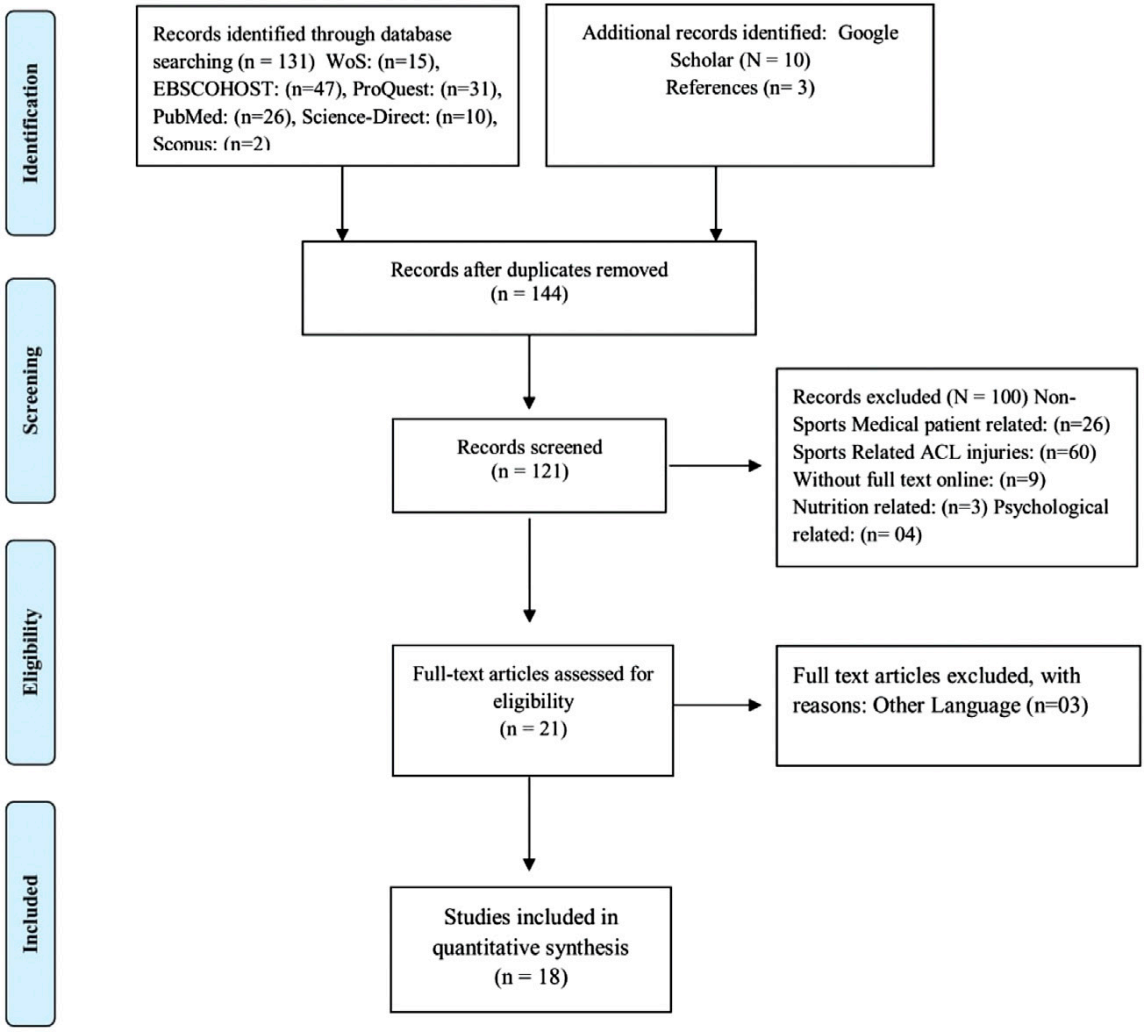
PRISMA flow chart of the study selection process.

The systematic review was conducted in compliance with the PRISMA statement’s criteria (Figure 1) (Moher et al., 2009), and the protocol was registered with INPLASY (Published: 30 October 2021; DOI: 10.37766/inplasy2021.10.0119). The PRISMA checklist is included in the article’s (see Figure 1).

### 2.2 Search strategy for the identification of studies

A systematic literature search was conducted using online: EBSCOHOST (SPORT-Discus), Google Scholar, ProQuest, Web of Science, PubMed, Mendeley and Science Direct. The search terms used were: “neuromuscular training,” neuromuscular exercise,” “cardiovascular fitness,” “physical endurance,” “physical conditioning,” “physical fitness,” “skill-related physical fitness,” “skill-related physical fitness,” “skill-related physical,” “skill related fitness,” “skill-related fitness,” quick change of direction, “agility,” “balance,” “power,” “speed,” “reaction time,” “coordination,” “health-related components,” health related components,” “health related physical,” “health-related fitness,” “health related physical fitness,” “muscular strength,” “cardiovascular endurance,” “aerobic endurance,” “muscular endurance,” “flexibility,” “body composition,” “sportswoman,” “sportsman,” “athlete” and “player”.

### 2.3 Study selection

The database search yielded 144 articles, and the screening process began by eliminating 23 duplicates. Then, 121 articles were separated for title and abstract; 100 articles were excluded related to medical patients, injuries, nutrition, and psychology, and 18 relevant full English articles were included. To determine whether the publications complied with the selection criteria, three independent reviewers initially reviewed the selected studies for title, abstract, and keywords. Each study must assess the impact of NT on various fitness components. Articles that satisfied the selection criteria and reports with unclear selection criteria in their abstracts (Figure 1).

### 2.4 Inclusion criteria

The following inclusion criteria were used to assess the NT effectiveness on performance: Athletes who participated in regular training routine/standardized protocol that was published in the English language; 2) male and female athletes are included; 3) NT interventions; 4) skills and health-related physical fitness components (agility, balance, coordination, speed, reaction time, power, flexibility, muscular strength, muscular endurance, body composition and cardiorespiratory endurance); 5) study design was limited to single-group randomized controlled trials (RCT) and non-randomized control trials (NRS) with two or more groups and pre-test and post-test designs.

### 2.5 Exclusion criteria

The excluding criteria for this study were: 1) studies that were in languages other than English; 2) abstracts, the conference published articles; 3) duplicate articles 4) sports psychology, nutrition-related and difficulty sleeping; 5) injury-related; and 6) exercise-related performance parameters or a full explanation of the athletes’ participation in sports or relevant training status.

### 2.6 Risk of bias and quality assessment

The adapted strengthening of the reporting of observational studies (STROBE) tool was used to assess the risk of bias in the selected studies (Lubans et al., 2010). Each of the following questions was scored with a 0 (absent or inadequately described) or 1 (available and thoroughly explained): 1) Does the study specify the method for choosing participants? 2) Are participants selected at random from the general population? 3) Did the study provide information on the sources and details of the procedure used to measure the benefits of physical training and whether the instrument was reliable? 4) Does the study provide the sources and details of the potential benefit assessment, and were all the methodologies reliable? 5) Is the power calculation reported in the study, and is the statistical approach used to test the hypothesis adequate? 6) Is the study’s total number of participants for each outcome measurement reported? 7) Is this figure account for at least 80% of the total sample? Each article received a score ranging from 0 to 6. Studies with a score of 0–2 were of low quality, a score of 3–4 was considered medium-quality, and a score of 5–6 was deemed high-quality. Apart from that, a score of 0–2 was considered a high risk of bias, a score of 3–4 was classified as medium risk, and a score of 5–6 was of low risk of bias (Table 1).

**TABLE 1:** Risk of bias assessment of the included studies.

Study	Criteria						Total score
	1	2	3	4	5	6	
Sahin et al. (2014)	1	0	1	1	0	1	4/6
Ahmed. (2015)	1	1	1	1	1	1	6/6
Borghuis et al. (2013)	1	1	1	1	1	1	6/6
McLeod et al. (2009)	1	0	1	1	1	1	5/6
Barber-Westin et al. (2010)	0	0	1	1	1	1	4/6
Pasanen et al. (2009)	1	0	1	1	1	1	5/6
Neto et al. (2006)	1	0	1	1	1	0	4/6
Dhawale et al. (2020)	1	0	1	1	1	1	5/6
Nunes et al. (2021)	1	0	1	1	1	1	5/6
Trajković & Bogataj, (2020)	1	0	1	1	1	1	5/6
Panagoulis et al. (2020)	1	1	1	1	1	1	6/6
Barber-Westin et al. (2015)	1	0	0	1	1	1	4/6
Ondra et al. (2017)	1	0	1	1	1	1	5/6
Pfile et al. (2016)	1	0	0	0	1	1	3/6
Kowalczyk et al. (2019)	1	0	1	1	1	1	5/6
Hydar Abbas et al. (2019)	0	0	1	1	1	1	4/6
Mendiguchia et al. (2015)	1	0	1	1	1	1	5/6
Canli. (2019)	1	1	1	1	1	1	6/6

### 2.7 Data extraction

Three independent reviewers extracted the data from studies that fulfilled the eligibility requirements. Journal of publication, publication year, title, authors, gender, age, number of subjects involved in the intervention and treatment, category of interventions, characteristics of the intervention study design, and outcome data were obtained from each study. Additionally, training frequency and training duration were extracted from the selected papers. It was assumed that conflicts in every aspect of the data collection were resolved through discussion between the three reviewers, who agreed with the final data reports.

## 3 Results

### 3.1 Study selection

The data selection is illustrated in (Figure 1). A total of 144 articles were identified through the electronic database search: EBSCOHOST (SPORT Discus) (41), PubMed (26), WOS (15), Mendeley (6), Scopus (2), ProQuest (31), Science-Direct (10), Google Scholar (*n* = 10), and additional relevant articles and previous related review reference lists (*n* = 3). After removing duplicates (121), the title and concepts were evaluated for eligibility. After the first 100 were eliminated based on title and abstract, the remaining 23 articles were examined. After reading, three studies from another language were deleted, leaving 18 articles that met the inclusion criteria and included in this systematic review.

### 3.2 Study quality assessment

The percentage of research that showed statistically significant associations was used to assess the scientific evidence while considering the possibility of bias (Lubans et al., 2010):

1. No association if less than 33% of the studies showed a significant relationship between variables.
2. Uncertain association if 34%–59% of studies showed a significant relationship between variables
3. If 60%–100% of the research found a substantial association, it may be a positive or negative association (in the same direction).
4. There is strong evidence of a positive or negative relationship. If 60%–100% of the studies identified a significant relationship between variables (in the same direction) and more than 59% of the studies have a low risk of bias (score ≥5) found a significant relationship. Each article received a score ranging from 0 to 6. None of the studies had a high risk of bias; studies with scores of three and four were classified as medium risk, while the low risk of bias studies had scores of five and 6 (see Table 1).

### 3.3 Review characteristics

There were 18 experimental groups with 725 people in the 18 studies. The details are as the following:

1. Soccer = 186 male players,
2. Basketball = 191 players (92 females 92 and 99 males),
3. Tennis = 57 players (41 females and 16 males),
4. Football = 234 players (222 females and 21 males),
5. Volleyball = 107 players (75 females and 32 males),
6. Cricket = 43 male players.

Age range: From age 10 years of group (Canli, 2019; Trajković and Bogataj, 2020) and up to 27 (Pasanen et al., 2009) experimental groups. Among the 18 studies, ten assessed males, five were exclusively females, while only three evaluated both genders (Table 3). Various forms of NT in different sports were applied in the 18 studies: Four studies focused on soccer players (Borghuis et al., 2013; Sahin et al., 2014; Kowalczyk et al., 2019; Panagoulis et al., 2020), six studies tested the effects of NT in a population of basketball players (McLeod et al., 2009; Ahmed, 2015; Pfile et al., 2016; Ondra et al., 2017; Canli, 2019), three studies tested volleyball players (Neto et al., 2006; Trajković and Bogataj, 2020; Nunes et al., 2021), two studies studied floorball players (Pasanen et al., 2009; Mendiguchia et al., 2015), and one study assessed cricket players (Hydar Abbas et al., 2019).

### 3.4 Interventions characteristics

The intervention period in the 18 studies ranged from 6–34 weeks (Table 2). The NT intervention program consisted of balance, plyometrics, and strength exercises.

**TABLE 2:** Population, Study design, interventions, and test project.

Study	Population	Study Design						
		Duration (Weeks)	Frequency	Design	Intervention	Main exercises	Training arrangement	Test Project
Sahin et al. (2014)	Young soccer players	Length:6-week/ Time:25 min	3 per week	Pre-post test	NT	Balance exercises	15-rep for both legs	Single leg- hop
Ahmed (2015)	Basketball players	Length: 8-week/ Time:60 min	3 per week	Pre-post test	NT	Muscular strength and endurance	3 sets 160 sec	Grip strength and vertical jump, push-up test and sit- up
Borghuis et al. (2013)	Elite young soccer players	Length:23-week/ Time 90 min	2 a week	Pre-post test	NT	Stability and agility exercise	20 s sitting boards, 3 sets	One-leg standing, balance, slalom sprint
McLeod et al. (2009)	Highschool basketball players	Length: 6-week/ Time: 70 min	2 per week	Pre-post test	NT	Functional strengthening, plyometrics, agility, balance training.	30 min,20min,10min,10min	Jumping rope, cone runs, and shuttle runs, single- and double-leg balance
Barber-Westin et al. (2010)	Junior tennis players	Length: 6-week/ Time: 90 min	3 per week	Pre-post test	NT	Jump training, Strength Agility Speed, Cardiovascular, Flexibility	20–25 s, 2 Sets of 6–16 reps, 2 Sets of 8 reps	Tuck jump, Medicine ball forehand, singles sideline–sideline, Iliotibial band Hip flexor
Pasanen et al. (2009)	Top level football	Length: 6 Months/Time: 25 min	3 per week	Pre-post test	NWP	Running, balance, jumping and strengthening exercises	Each exercise type taking 5–7 min	Static jump, countermovement jump, jumping over a bar, standing on a bar and figure-of-eight running.
Neto et al. (2006)	Athletes, child and junior high	Length: 34-week/ Time: NR	1.8 (± 0.4) per week	NR	NT	Assess the vertical jump, maximal Oxygen consumption	4 phases	1,000 meters running speed
Dhawale et al. (2020)	Basketball Players	Length: 6-week/ Time: NR	4 per week	Pre-post test	DNE	Illinois agility test and Medicine ball throw test	10 reps, 10 rep,10rep, 10 jumps	Catching & throwing weighted ball, Low oblique sit exercise, Repetitive jumping
Nunes et al. (2021)	Volleyball players	Length: 12-week/ Time:60 min	2 per week	Pre-post test	INT	Countermovement vertical jump height	2 per 10 reps, 2 per 10 rep, 3set 35sec, 2set 15sec	Squatting Stairway of agility, Lunge Vertical line jumps Balance on the foot tip

Study	Population	Study Design						
		Duration (Weeks)	Frequency	Design	Intervention	Main exercises	Training arrangement	Test Project
Trajković & Bogataj, (2020)	Volleyball players	Length: 8-week/ Time: 90 min	2 per week	Pre-post test	NT	Plank, VJ, medicine ball throw	60–90 s,60-90 s, 2 sets of 8–12 rep, 8–12 rep, 2 rep, 2 sets	Side plank, Plank, Hurdle jumps, Agility ladder various patterns, Backward lunges with Med Ball
Panagoulis et al. (2020)	Soccer players	Length: 8-week/ Time: 20 min	3 per week	Pre-post test	INT	CS, hamstrings eccentric strength, dynamic stability body mass exercises	5 sets 16 set, 3 sets of 10-12 rep, 3 sets 8 rep	Hip, knee dominant exercises, squat, single-leg box step-ups
Barber-Westin et al. (2015)	Junior tennis players	Length: 6-week/ Time: 90 min	3 per week	Pre-post test	NPT	Dynamic warm-up, jump training, tennis specific agility and hitting drills	2-4 reps, 2 sets of 8 reps, 2 sets, 20 sec, 10 reps	Two single leg hop tests, baseline and service box speed and agility tests, abdominal endurance test
Ondra et al. (2017)	Elite youth basketball Players	Length: 20-week/ Time: 20 min	3 per weekly	Pre-post test	N and proprioceptive training	Balance and sport-specific exercises	Each 20 min	Balance, strength, plyometric, agility
Pfile et al. (2016)	Basketball players	Length: 6-week/ Time: 30 min	3 per weekly	Pre-post test	NT	Dynamic warm-up followed by exercises	30 min	Isometric strength, plyometric
Kowalczyk et al. (2019), Hydar Abbas et al. (2019), Mendiguchia et al. (2015)	Top professional soccer players	Length: 3 weeks/ Time: 20 min	2 per week	Pre-post test	NT	Basic Stability, Advanced Stability, and Advances Dynamic Stability	5 min	Running, dynamic stretching, balance, agility, coordination, neuromuscular control, and plyometric training.
							10 min	
							5 min	
Canli. (2019)	Asymptomatic sub-elite cricket players	Length: 12-week/Time: NR	3 per week	Pre-post test	NT	Mild intensity running, flexion and extension exercises	5 -10 min, 3 set10 rep	Dynamic rhythmic stabilization drills, Speed radar gun
Trajković & Bogataj, (2020), Panagoulis et al. (2020), Barber-Westin et al. (2015)	Amateur football	Length: 7-week/ Time: 90 min	2 per week	Pre-post test	NT	Plyometric, strength exercises	5 min	Low-pace (∼10 km/h) running, lower limb muscle stretching, sprint-specific drills
							3 min	
							2 min	
Ondra et al. (2017)	Basketball players	Length: 8-week/ Time: 90 min	2 per week	Pre-post test	NT	Isometric squat, plank, Isometric push up	15 sec	Old + 6 squat jumps Hold, hold + 6 push up
							30 sec	
							6 sec	

Abbreviation—N, neuromuscular; NT, neuromuscular training; NWP, Neuromuscular warm-up program; DNE, dynamic neuromuscular exercise; INT, integrative neuromuscular training; NPT, neuromuscular performance and training; Sec, second; Min, Minute; Rep, Repetition; CS, Core stability; VJ, Vertical jump; NR Not reported.

### 3.5 Outcomes

#### 3.5.1 Effect of neuromuscular training on balance

Nine studies assessed balance-related outcomes: young male soccer players; (Borghuis et al., 2013; Sahin et al., 2014; Kowalczyk et al., 2019), basketball players; (Neto et al., 2006; Pfile et al., 2016; Ondra et al., 2017), football players; (Pasanen et al., 2009), tennis players; (Barber-Westin et al., 2010; Barber-Westin et al., 2015), and volleyball players; (Trajković and Bogataj, 2020). The dynamic balance was examined in a quiet unipedal posture and positively influenced postural stability for dominant and non-dominant limbs (Ondra et al., 2017). Static and dynamic balance resulted in fewer Balance Error Scoring System (BESS) errors in the trained group at the posttest compared with their pretest and the control group (*p* = 0.003) and in Star Excursion Balance Test (SEBT) compared with the controlled group after the posttest (*p* < 0.05) (McLeod et al., 2009). Three studies assessed how football, tennis, and soccer players balance on similar surfaces. During the follow-up, significant changes in static and dynamic balance were observed in the intervention group compared to the control group players (Neto et al., 2006; Barber-Westin et al., 2010; Sahin et al., 2014). In one study, the single-leg triple crossover had significant improvement after the intervention, but in the single-leg hop, improvement was only (29%) and showed no significant effect (Barber-Westin et al., 2015).

#### 3.5.2 Effect of neuromuscular training on speed

The effect of NT on speed performance was reported in four out of the 18 studies selected in this systematic review (Schneider et al., 2006; Barber-Westin et al., 2010; Sahin et al., 2014; Barber-Westin et al., 2015). The speed evaluation tools included the figure-of-eight running tests (Pasanen et al., 2009), baseline and service box speed tests; (Barber-Westin et al., 2010; Barber-Westin et al., 2015), 5-m sprint tests; (Mendiguchia et al., 2015; Canli, 2019), and functional throwing performance index (FTPI) test (Hydar Abbas et al., 2019). The subjects included top-level floorball players (Pasanen et al., 2009; Mendiguchia et al., 2015), professional junior male and female tennis players (Barber-Westin et al., 2010; Barber-Westin et al., 2015), moderately-trained football players (Sahin et al., 2014) and sub-elite cricket players (Hydar Abbas et al., 2019).

Two studies showed improvement (69%–76%) in baseline and service box speed tests among professional junior male and female tennis players, regardless of gender (Barber-Westin et al., 2010; Barber-Westin et al., 2015). A neuromuscular warm-up program dramatically improved the sideways jumping speed of floorball players (Pasanen et al., 2009; Mendiguchia et al., 2015). However, in another study, throwing speed was not significantly improved 6 weeks post-withdrawal of the NT, but after 12 weeks of neuromuscular training, significantly improved speed (*p* < 0.001) (Hydar Abbas et al., 2019) (Table 3). Furthermore, a slight improvement had a small effect size (ES = 0.32) in a 5-m sprint, and an effect size (0.27) in a 20-m sprint was evident in the experimental group, while the control group exhibited minor sprinting performance impairments (Mendiguchia et al., 2015; Canli, 2019).

**TABLE 3:** Group, Main outcomes, and participants characteristics.

Study	Group	Main Outcomes	Participants Characteristics		
			N (I/C)	Sex	Age (years)
Sahin et al. (2014)	NT	Implemented short term training improvements in the parameters investigated in this study. However, it was only effective on right leg hop distance.	32	M	(EG=17) (age= 15 years, height =1.72± 5.98 m, weight= 62.56 ± 6.12 kg), CG=15 (age=14 years, height =1.70±5.32 m, weight=58.6 ± 7.24 kg)
Ahmed (2015)	NT	Significant improvement, but the greater percentage of change is found in NMT group. Improvement in musculoskeletal fitness was ranged between 17% to 47% for NMT group versus 5% to 13% for CON, while ranged between 18% to 30% for NMT group versus 10% to 17% for CON group in skills performance.	24	M	NMT=12 (age=18.04±0.68 years, height = 179.91±1.67 cm, body mass=67.58±1.31 kg, CON=12 (age= 18±0.47 years, height =179.58 ± 1.62 cm, body mass = 67.41±0.99 kg
Borghuis et al. (2013)	NT	Improvements in 20-m sprint, agility performance, CJH and NC of the lower-limb as assessed via single-leg squat.	90	M	EG age= 14.3 (1.7) years, weight 52.3 (12.1), Kg, Hight 1.64 (0.10) CG, age=14.2 (1.7), weight 52.6 (11.2) Hight 1.64 (0.13)
McLeod et al. (2009)	NT	The study demonstrates that an NTP can increase the balance and proprioceptive capabilities of female high school basketball players and that clinical balance measures are sensitive to detect these differences.	62	F	TG, n=37 (age=15.6 ± 1.1 years, Hight 170.7 ± 6.8cm, Mass 58.9 ± 5.9 Kg,) CG, n =25 (age=16.0 ± 1.3 Hight 171.5 ± 8.1cm, Mass 62.3 ±7.6Kg)
Barber-Westin et al. (2010)	NT	The improvements were noted for both baseline tests, because 67% improved the forehand test score and 80% improved the backhand score.	15	M=5 F=10	5 boys, mean (age 13.0 6 1.5 years, range, 11–16 years)
Pasanen et al. (2009)	NWP	Improved the floorball players’ sideways jumping, speed and static balance.	222	F	Intervention (n=119, age= 24.2 (4.2) year, Hight (m) 165.4 (4.9), wight (Kg) 62.6 (8.5), BMI (kg/m2) 22.8 (2.7), Control (n=103, age= 23.3 (5.3) year, Hight (m) 167.1 (5.6), wight (Kg) 63.7 (7.5), BMI (kg/m2) 22.8 (2.3)
Neto et al. (2006)	NT	The performance test for the blockage range showed a significant difference of p < 0.01 comparing the third (2.56 ± 0.1 m) to the second assessment (2.54 ± 0.1 m), and p < 0.05 comparing the fourth (2.56 ± 0.11 m) to the second assessment (2.54 ± 0.1 m).	9	F	Age=11.3 (± 1.0) years old
Dhawale et al. (2020)	DTE	There is significant effect of DNT on explosive arm strength and agility in basketball players.	60	M=30 F=30	EG-30, Age= 21.37 ± 2.12 years, CG-30, Age= 22.9 2.41 years
Nunes et al. (2021)	INT	Enhance jumping performance in young volleyball players, and that this type of neuromuscular program may be particularly beneficial for youth with limited fundamental motor skill performance.	32	M=19 F=13	INT (age: 13.16 ± 0.4 years; body mass: 55.36± 12.1 kg; and height: 161.16± 6.4 cm), CON (age: 12.86± 0.7 years; body mass: 51.86± 13.6 kg; and height: 160.1± 610.7 cm).

Study	Group	Main Outcomes	Participants Characteristics		
			N (I/C)	Sex	Age (years)
Trajković & Bogataj, (2020)	NT	The NMT promoted significant gains in motor competence and physical performance in youth female volleyball players.	66	F	EG-34 (age, 11.12 ± 0.68 years; height 158.28 ± 8.07 cm; weight 47.83 ± 8.97 kg) CG- 34 (age, 10.96 ± 0.75years; height 157.37 ± 10.21cm; weight 48.59 ± 13.46 kg)
Panagoulis et al. (2020)	INT	INT program may induce positive adaptations in performance of early-adolescent soccer players during in-season training, suggesting that INT may be an effective training intervention for this age group.	28	M=14 F=14	EG (Age= 11.2 6 0.5 years, CG Age=11.4 6 0.57 years)
Barber-Westin et al. (2015)	NPT	Significant improvements and large-moderate effect sizes were measured for speed, agility, dynamic single-leg balance, and abdominal endurance.	42	F=31 M=11	(Mean age, 14 ± 2 years)
Ondra et al. (2017)	N and Proprioceptive training	The specific proprioceptive and neuromuscular training had a positive effect on postural stability for both the dominant and non-dominant limb in basketball players.	21	M	(EG=10: *n* = 10, age 17.3 ± 1.3 years, height 189.4 ± 9.4 cm, weight 77.9 ± 7.8 kg. CG: *n* = 11, age 16.5 ± 1.8 years, height 187 ± 8.2 cm, weight 77.8 ± 13.1 kg).
Pfile et al. (2016)	NT	Improved landing mechanics and dynamic balance over a 9-mo period in women’s basketball players. NC adaptations can be retained without an in-season maintenance program.	11	F	(Age 19.40 ± 1.35 y, height 178.05 ± 7.52 cm, mass 72.86 ± 10.70 kg)
Kowalczyk et al. (2019)	NT	NT can be effective for postural control improvement in professional male soccer players within a short period of time. Leg dominance does not affect one-leg stability.	36	M	(EG=16 Age=24.0 ± 5.0, 18.0 ÷ 37.0 years, Hight (Cm) 182.0 ± 8.1, 171.0 ÷ 196.0 Body mass (kg) 77.2 ± 7.4, 65.8 ÷ 91.3, (CG=20 Age= 22.0 ± 4.6 18.0 ÷ 29.0 years, Hight (Cm) 179.0 ± 7.0 174.0 ÷ 189.0 Body mass (kg) 75.6 ± 6.2 62.1 ÷ 85.5
Hydar Abbas et al. (2019)	NT	Significant improvement in throwing accuracy (p <0.001) and speed (p <0.001) after 12 weeks of NT. Six weeks post-withdrawal of the NT on throwing accuracy was not significant (p=0.117), However, speed was sustained (p=0.013).	43	M	(Age: 20.4 ± 2.03 years; BMI: 21.4)
Mendiguchia et al. (2015)	NT	NT program can in duce positive hamstring strength and maintain sprinting performance, which might help in preventing hamstring strains in football players.	12	M	(N=31 the EG (22.2 ± 4.5 years; 70.7 ± 9.0 kg; 174.6 ± 6.1 cm), n=29 CG (21.9 ± 2.9 years; 71.4 ± 7.0 kg; 176.9 ± 5.8 cm)
Canli. (2019)	NT	Significant differences revealed in favor of the intervention group in agility, back strength, long jump, flexibility, and balance (p<0.05).	24	M	EG (n = 12, age = 10.7 ± 0.75 years, body height = 148.9 ± 9.14 cm, body weight = 40.8 ± 11.54 kg, CG (n = 12, age = 10.8 ± 0.68 years, body height = 149.8 ± 7.71 cm, body weight = 44.4 ± 8.04 kg

Abbreviation—NT, neuromuscular training; NWP, Neuromuscular warm-up program; DNE, dynamic neuromuscular exercise; INT, integrative neuromuscular training; NPT, neuromuscular performance and training; CJH, countermovement jump height; NC, neuromuscular control.

#### 3.5.3 Effect of neuromuscular training on agility

Agility was evaluated among young soccer players (Borghuis et al., 2013; Dhawale, 2020; Panagoulis et al., 2020), football players; (Barber-Westin et al., 2010; Barber-Westin et al., 2015), and volleyball players (Trajković and Bogataj, 2020), slalom sprint test (Borghuis et al., 2013), agility drills (Barber-Westin et al., 2010), a figure-of-eight running test (Pasanen et al., 2009), the Illinois agility test (Dhawale, 2020), and a *t*-test (Trajković and Bogataj, 2020). The NTG demonstrated a significant decrease in time for the modified *t*-test (*p* = 0.001, moderate ES), whereas the CON displayed only a small decrease (ES = 0.3%, 0.9% change) (Trajković and Bogataj, 2020) and another study results also showed a small effect size on *t*-test (0.49) (Canli, 2019)**.** In Table 3 one study determined the effect sizes (0.29) for results after the intervention was small in the slalom sprint test (Borghuis et al., 2013). Other studies reported significantly improved agility in players post-treatment (Pasanen et al., 2009; Barber-Westin et al., 2010; Dhawale et al., 2020; Panagoulis et al., 2020).

#### 3.5.4 Effect of neuromuscular training on muscular power and endurance

Muscular power and endurance were evaluated in football players (Pasanen et al., 2009), volley ball players (Nunes et al., 2021), tennis players (Barber-Westin et al., 2015). The static jump and the countermovement jump assessed the maximal muscular power of the lower extremity extensor muscles (Pasanen et al., 2009). After 6-week intervention, a significant difference was found between pretest (Mean = 25.6) and posttest (Mean = 26.91) of vertical jump averages (t 11) = −2,385, *p* < 0.05). Calculated effect size of (d = −0.36) shows that this difference is medium (Nunes et al., 2021). The abdominal endurance test enhances core endurance with large-moderate effect size (1.20–0.95) in junior tennis players (Barber-Westin et al., 2015) (Table 3).

## 4 Discussion

This systematic review provides a holistic perspective concerning the effects of NT on athletes’ physical fitness and its implications for athletes aiming to improve their fitness. The findings suggest that NT can help athletes enhance their physical fitness (balance, agility, speed, muscular power, muscular endurance, and coordination), but there is a lack of data in the reaction time assessment. The participants (age, gender, and types of athletes) and physical fitness components evaluated in the articles reviewed varied significantly. The non-significant effect of NT was observed in participants of varying ages (18–36 years) (Barber-Westin et al., 2010; Barber-Westin et al., 2015; Dhawale et al., 2020). Adolescent athletes were the focus of most of these intervention research, and the favorable impacts were mainly evident in terms of skills in subjects under 18 years old (McLeod et al., 2009; Sahin et al., 2014; Trajković and Bogataj, 2020).

This study outlined each exercise, the required performance, and the duration of each intervention session with in-depth instructional material. Table 2 demonstrates the number of training exercises completed by intervention groups and provides brief descriptions of the practices completed. Agility and muscular power are essential skills for most sports (Pasanen et al., 2009; Barber-Westin et al., 2015; Panagoulis et al., 2020). The conventional definition of agility is the quickness in directional movements. (Panagoulis et al., 2020). Both male and female subjects who participated in the 6-week NT program exhibited positive outcomes in terms of agility, with no significant difference between genders (Barber-Westin et al., 2015). Therefore, NT is a potentially sustainable method for improving agility despite previous research outcomes.

Another study found that an 8-weeks INT program increased the performance of adolescent players during the season, revealing the suitability of this intervention for this age group. Only one study mentioned muscle power. Moreover, after the 6-month intervention, static jumps, countermovement leaps, jumping over a bar, and standing on a bar were all part of the performance evaluations (Pasanen et al., 2009). The athletes showed significant improvements in jumping over a bar and the number of leaps in 15 s post-treatment (Pasanen et al., 2009). Besides, the exercises were also recommended for weekly floorball player training.

Many studies have highlighted the role of the NT intervention in enhancing athletes’ performance (Barber-Westin et al., 2010; Barber-Westin et al., 2015; Panagoulis et al., 2020) (Table 3). Specific NT programs have different aspects to focus on, such as strength, speed, skills, balance, and jumping exercises with several variations to make them more effective. Some programs were conducted at low intensity because of the athletes’ capabilities. Some studies compared the participants’ physical fitness in pre-test and post-test and reported that athletes who had NT significantly increased performance compared to the non-treated group (Borghuis et al., 2013; Sahin et al., 2014; Barber-Westin et al., 2015). Other studies have proven that athletes who underwent dynamic NT significantly improved their time and distance and showed a positive relationship between them and improvements (Barber-Westin et al., 2010; Canli, 2019; Dhawale et al., 2020; Panagoulis et al., 2020; Trajković and Bogataj, 2020; Nunes et al., 2021).

A month-long circuit-type INT program in a small group improved their daily calorie expenditure over intake, decreased body fat mass and boosted strength and cardiovascular performance. In the following months, the outcome implied the need to integrate neuromuscular strength training programs to improve performance, such as plyometrics and training-induced gains. These speed improvements may be related to increased neuromuscular activation, ground contact time, and muscle-tendon unit stiffness (Barber-Westin et al., 2015). In addition, two out of the 18 studies evaluated static balance, while four studies assessed the dynamic balance in athletes. These investigations confirmed that participants in an NT program increase their static and dynamic balance after completing the training program (McLeod et al., 2009; Pasanen et al., 2009; Barber-Westin et al., 2015; Pfile et al., 2016; Nunes et al., 2021). Meanwhile, a study that compared a soccer-specific NT program to a twice-weekly, 23-weeks balance training program and dynamic movement training exercise on a stability ball found that the soccer-specific NT program significantly improved single-limb static balance compared to other training programs (Borghuis et al., 2013).

Furthermore, this review demonstrates that NT programs can help athletes improve their performance while simultaneously preventing injuries. The NT focuses on activities that support the brain by training nerves and muscles to enhance the connection between the athlete’s body and subconscious mind. This coordination helps athletes increase their balance, leap, agility, speed, strength, muscular power, muscular endurance, coordination, and cardiorespiratory endurance by improving the brain’s ability to control limb movements. Speed and strength have a considerable influence on the development of physical fitness. According to the review, players must increase strength training to enhance their abilities and remain competitive in their respective fields.

One study demonstrated a substantial difference in the advantages of a field-based, 7-week NT exercises program that combines posterior thigh eccentric-biased muscular strength with lower-limb plyometrics with a horizontal focus. Consequently, the players significantly increased lower limb strength (Sahin et al., 2014; Dhawale et al., 2020). Another study compared a soccer-specific NT program to a twice-weekly 23-weeks balance training program. Dynamic movement training exercises significantly improve single-limb static balance on a stability ball (Yoo et al., 2010; Trajković and Bogataj, 2020). Therefore, the NT program is a valuable technique for improving strength, agility, jumping ability, balance, and speed following these review studies.

## 5 Limitations

Overall, this study found substantial evidence regarding the positive impacts of numerous NT methods on athletes’ physical fitness. However, this review does have some limitations. First, there were limited studies on muscular strength and explosive power. No study has seen the effect of NT on Coordination, reaction time, cardiovascular fitness, flexibility, body composition and cardiorespiratory fitness. Limited studies showed the impact of NT on female players. Most studies used a short intervention length due to did not see the large effect of NT on fitness components. Only three research reported mixed-gender athletes. Gender is a crucial factor because of the different standards in measuring physical fitness components that could significantly impact the final results. Furthermore, studies also completely neglected the impact of temperature, time, and other variables on the athletes’ physical fitness.

## 6 Conclusion and recommendations

In conclusion, NT positively impacts athletes’ physical fitness (balance, agility, speed, muscular power, muscular endurance) and improves the performance of motor muscles, which speeds up the subcortical control system. According to this assessment, NT should focus on performing exercises that support brain training of the muscles and nerves to improve communication by coordinating the athlete’s body and subconscious mind. A successful fitness training schedule for players should begin in the preseason, last at least 12 weeks, and continue in-season frequently (3 times a week). Essential training components include balance, proprioception, plyometrics, strength training, and instructional feedback on proper technique. The effects of NT on other physical fitness components, including coordination, reaction times, flexibility, cardiovascular fitness, cardiorespiratory fitness, and body composition, should be further studied. NT is essential for athletes of all ages and genders. Furthermore, RCTs with high methodological quality should be conducted on female sporting populations of multiple age groups and athletic levels to understand further the effectiveness of particular exercise interventions on physical fitness components. Further investigation is also needed into the impact of NT on the physical fitness required in other sports like handball, hockey, judo, badminton, kabaddi, wrestling, badminton, and athletics.

## 7 Practical application

Players’ muscular power, balance and agility were influenced by slower and faster directions which were essential for player performance. Therefore, in the NT program, the target fitness trainers should be the slower and quicker change direction, balance, and muscular strength. Furthermore, coaches should conduct a neuromuscular training length of 12 weeks, 3 times per week, and a 90-min training duration. A dynamic warm-up, plyometric and jump training, and strength training are all part of the program (lower extremity, upper extremity, core); young players who participate in this program seem to benefit from increased speed, agility, abdominal endurance, single-leg function, and balance.

## Data Availability

The original contributions presented in the study are included in the article/supplementary material, further inquiries can be directed to the corresponding authors.
